# Pulse Wave Velocity and Blood Pressure Variability as Prognostic Indicators in Very Elderly Patients

**DOI:** 10.3390/jcm12041510

**Published:** 2023-02-14

**Authors:** Alejandro de la Sierra, Cristina Sierra, Marcos Murillo, Tomasso F. Aiello, Aina Mateu, Pedro Almagro

**Affiliations:** 1 Department of Internal Medicine, Hospital Mútua Terrassa, University of Barcelona, 08221 Terrassa, Spain; 2 Department of Internal Medicine, Hospital Clínic, University of Barcelona, 08036 Barcelona, Spain; 3 Emergency Department, Hospital Mútua Terrassa, University of Barcelona, 08221 Terrassa, Spain

**Keywords:** arterial stiffness, pulse wave velocity, blood pressure variability, mortality, very old population, decompensated chronic conditions

## Abstract

There is scarce evidence for the prognostic importance of hemodynamic measures, such as blood pressure (BP), BP variability, and arterial stiffness, in the very elderly population with advanced chronic conditions. We aimed to evaluate the prognostic importance of 24 h BP, BP variability, and arterial stiffness in a cohort of very elderly patients admitted to the hospital due to a decompensated chronic disease. We studied 249 patients older than 80 (66% women; 60% congestive heart failure). Noninvasive 24 h monitoring was used to determine 24 h brachial and central BP, BP and heart rate variabilities, aortic pulse wave velocity, and BP variability ratios during admission. The primary outcome was 1-year mortality. Aortic pulse wave velocity (3.3 times for each SD increase) and BP variability ratio (31% for each SD increase) were associated with 1-year mortality, after adjustments for clinical confounders. Increased systolic BP variability (38% increase for each SD change) and reduced heart rate variability (32% increase for each SD change) also predicted 1-year mortality. In conclusion, increased aortic stiffness and BP and heart rate variabilities predict 1-year mortality in very elderly patients with decompensated chronic conditions. Measurements of such estimates could be useful in the prognostic evaluation of this specific population.

## 1. Introduction

The prognostic importance of several hemodynamic parameters, such as blood pressure (BP) (both peripheral and central) [1,2], BP variability [3], and pulse wave velocity [4,5], as markers of arterial stiffness is very well established in the general population, as well as in individuals with cardiovascular risk factors and diseases.

Advanced age modifies the relationship between other cardiovascular risk factors and prognosis. An analysis of a subgroup of patients older than 80 years who participated in clinical trials of antihypertensive treatment in the elderly suggested an inverse relationship between BP and mortality [6]. In contrast, more recent data from the Hypertension in the Very Elderly Trial [7] and the Systolic Blood Pressure Intervention Trial [8] indicated that BP reduction had a positive impact in very old patients. Current hypertension guidelines recommend an individualization of antihypertensive treatment in patients older than 80, depending on frailty and general health status [9].

The population of very old patients with multiple comorbidities and physical functional impairment is prevalent in general hospitals [10]. They are usually admitted to general wards due to decompensation of one or several chronic conditions. In addition, those admitted for other reasons frequently suffer from stressful situations, such as infection, trauma, or surgery, which, in turn, alter the function of such chronic conditions. As a result, they are prone to suffer decompensations. Hemodynamic changes and significant BP oscillations are frequent during hospitalization, and, in some cases, are also associated with the use of short-acting drugs modifying BP, which promote a further increase in short-term BP variability.

There is very scarce evidence regarding the prognostic impact of such hemodynamic parameters in very old patients hospitalized with chronic disease decompensations. A previous study examining peripheral and central BP, as well as pulse wave velocity, did not find any association with 1-year mortality [11]. Other studies have found a correlation between arterial stiffness and mortality in old patients (usually older than 65 years) [12,13] or in those with heart failure with preserved ejection fraction (mean age 75) [14]. The prognostic impact of BP variability is also controversial in such populations [15,16,17,18,19].

Given the scarce information on this population of very old, hospitalized patients, the aim of the present study was to assess the prognostic impact on 1-year mortality of several hemodynamic measures, including BP (both peripheral and central), short-term BP variability, and measures of arterial stiffness, in patients older than 80 admitted to the hospital due to a decompensation of a chronic condition.

## 2. Patients and Methods

### 2.1. Study Design

This prospective cohort study included 249 patients aged >80 years who were hospitalized in conventional wards at the internal medicine departments of 1 of the 2 participant hospitals due to a decompensation of a chronic condition. A mini-mental scale examination with a final score of 24 or more was required for inclusion. The local Institutional Ethic Committees approved the study protocol. Written informed consent was obtained from all participants. The investigation conforms to the principles outlined in the Declaration of Helsinki.

Demographic, anthropometric, and clinical characteristics were recorded at admission from all participants and the following data were obtained: age, sex, weight, height, smoking habit, hypertension (BP ≥ 140 and/or 90 mmHg, or use of antihypertensive treatment), diabetes (2 or more fasting plasma glucose determinations ≥ 7.0 mmol/L, or use of antidiabetic treatment), and previous history of cardiovascular disease (including cerebrovascular disease, coronary heart disease, heart failure, or symptomatic ischemic peripheral vascular disease). Moreover, physical functional status was assessed by the Barthel Index and multimorbidity by the Charlson Index, without age adjustment.

### 2.2. Measurement of Blood Pressure, Heart Rate, Blood Pressure and Heart Rate Variabilities, and Pulse Wave Velocity

All measurements were performed by means of a noninvasive automated oscillometric device (Mobil-O-Graph PWV, IEM, Stolberg, Germany), with the inbuilt ARCSolver method used for both peripheral and central 24 h BP assessment. The system has been validated for brachial BP measurement, according to the European Society of Hypertension International Protocol [20]. Moreover, the methodology for aortic pulse wave velocity (aPWV) estimation has been previously reported and validated against invasive methods [21,22]. The monitor was placed on patients during the first 2 days after admission, starting between 08:00 A.M. and 10:00 A.M. The appropriate cuff was selected according to the patient’s arm circumference. BP (both peripheral and central), heart rate, and aPWV were measured automatically at 15 min intervals over 24 h. All subjects included in this study had recordings of good technical quality (at least 70% of readings were valid, and at least 1 measurement was taken per hour). Otherwise, 24 h measurements were immediately repeated.

Blood pressure and heart rate variabilities were estimated by calculating standard deviations (SDs) for 24 h systolic and diastolic peripheral and central BP, as well as heart rate. BP variability ratio (BPVR) was calculated as the ratio between systolic and diastolic 24 h SD, as previously reported [23].

### 2.3. Assessment of Mortality and Readmission

Electronic medical records were reviewed 1 year after hospital discharge in order to assess vital status, cause of death, as well as readmissions. In a few patients, the electronic medical record did not contain any annotation after hospital discharge. In such cases, telephone contact was established to assess the outcome. The primary outcome was total mortality at 1 year after discharge. Secondary endpoint was the combination of death or readmission 1 year after discharge.

### 2.4. Statistical Analysis

Data are presented as frequencies and percentages for qualitative variables and as mean ± SD or median (interquartile range) for quantitative variables. Differences in study variables between groups were assessed with the Pearson’s χ^2^ for qualitative variables and Student’s *t*-test or Mann–Whitney *U* test for quantitative data.

The associations between measurements of BP, heart rate, BP and heart rate variabilities, aPWV, and both the primary and secondary outcomes were summarized with hazard ratios (HRs) and their 95% CI for 1 SD change, estimated by Cox models. Three Cox models were constructed. Model 1 was unadjusted. Model 2 was adjusted for age, sex (male/female), obesity (yes/no), diabetes (yes/no), previous cardiovascular disease (yes/no), diagnosis of congestive heart failure decompensation (yes/no), concomitant use of beta blockers (yes/no), physical functional status (Barthel Index), and comorbidities (Charlson Index). Model 3 (BP and heart rate variabilities and arterial stiffness) was additionally adjusted for the correspondent 24 h systolic BP (aPWV, systolic SD, and BPVR), diastolic (diastolic SD) BP, or 24 h heart rate (heart rate variability).

We assessed consistency in the results according to age (≤85 and >85 years), sex (men and women), diabetes mellitus (yes and no), physical functional status (Barthel Index < 70 or ≥70), and main cause of hospitalization (congestive heart failure or others).

SPSS for Windows version 25.0 software (IBM, Armonk, NY, USA) was used for statistical analysis.

## 3. Results

A total of 249 patients fulfilled the inclusion criteria and were included in this study. Main admission diagnosis was congestive heart failure in 149 patients (59.8%), chronic obstructive pulmonary disease in 60 (24.1%), chronic kidney disease with superimposed acute kidney injury and/or infection in 26 (10.4%), and other conditions in 14 (5.6%). All patients were available for follow-up at one year by reviewing electronic medical records (240) or through telephone contact (9).

During the year of follow-up, 72 patients (28.9%) died. Progression to heart failure was the most frequent cause of death (42 patients; 58.3%), followed by infection (12 patients; 16.7%) and progression of renal disease (9 patients; 12.5%). The number of patients requiring one or more hospitalizations during follow-up was 123 (49.4%). The combination of death and readmission was present in 159 (63.9%) patients.

Table 1 shows differences in clinical characteristics as well as BP estimates between patients who died or who remained alive at the end of the 1-year follow-up. Patients who died during follow-up were older, had more comorbidities as assessed by the Charlson Index, and had a poorer functional status, as defined by a lower Barthel Index (65 vs. 85; *p* < 0.001). No differences were observed in mean values of 24 h systolic or diastolic BP, brachial or aortic, or heart rate. Systolic BP variability (SD of 24-h BP), both brachial and aortic, was significantly higher in patients who died compared with those remaining alive at 1-year follow-up, while no differences were found in diastolic BP variability. Patients who died showed significantly lower values of heart rate variability compared to those alive at the end of the follow-up (5.9 ± 2.8 vs. 7.3 ± 3.4 bpm; *p* = 0.003). Aortic PWV was significantly elevated in patients who died during follow-up in comparison to those remaining alive (13.8 ± 1.0 vs. 13.1 ± 1.0 m/s; *p* < 0.001). BPVR was also higher in patients who subsequently died.

Table 2 shows hazard ratios for 1 SD change in measured hemodynamic parameters in relation to mortality. In models adjusted for clinical confounders, none of the BP estimates (brachial or aortic) showed significant relationships with mortality. In fully adjusted models (clinical confounders and corresponding BP), brachial systolic BP variability (HR: 1.38; 95%; CI: 1.06–1.80), heart rate variability (HR: 0.68; 0.48–0.95), aPWV (HR: 3.30; 2.20–4.93), and brachial BPVR (HR: 1.31; 1.06–1.62) were all associated with 1-year mortality. These results were consistent in subgroups of patients depending on age (≤ or >85 years), sex (males or females), diabetes (yes or no), physical functional status (Barthel Index < or ≥70), and cause of admission (congestive heart failure vs. others) (Supplementary Tables S1–S5).

When compared to patients alive and free of readmissions, those who died or suffered at least one more hospitalization were less frequently obese and less frequently had a previous hypertension diagnosis. No differences were observed in terms of age, functional status, or comorbidities. Moreover, there were no differences in 24 h BP or heart rate, BP or heart rate variabilities, or aPWV. Brachial systolic BPVR was significantly higher in the group of patients who died or were readmitted during the year of follow-up (Table 3).

Table 4 shows HR of the studied parameters in relation to the aforementioned secondary endpoint. Only aPWV (HR: 2.11; 1.49–3.00) and brachial BPVR (HR: 1.19; 1.03–1.38) were significantly associated with the combined endpoint of mortality or readmission, while BP, heart rate, and BP or heart rate variabilities showed no significant association.

## 4. Discussion

This study shows that increased arterial stiffness and BP variability are predictors of 1-year mortality in very elderly patients admitted to the hospital due to a decompensation of a chronic condition. For each SD increase in aPWV, 1-year mortality increased 3.3-times in models adjusted for clinical confounders and 24 h BP. SD of 24 h systolic BP and BPVR (the ratio between SD of systolic and diastolic BP) also increased the risk of 1-year mortality by 38% and 31%, respectively, in fully adjusted models. The absolute level of BP (either brachial or aortic) did not show any relationship with 1-year mortality.

The relationship between BP and mortality is well established in the general population [1,24]. Moreover, some studies have suggested that central or aortic BP, measured by pulse wave analysis, displays a better relationship with cardiovascular outcomes compared to brachial BP measurements [2]. However, this relationship is attenuated with advanced age and controversial results have been obtained regarding the effect of BP lowering with antihypertensive treatment in very elderly individuals. An analysis of patients older than 80 who participated in clinical trials of antihypertensive treatment suggested that the benefit of preventing cardiovascular events was not translated into reductions in mortality [6]. In fact, such analysis found a non-significant increase (6%) in total mortality in patients receiving active treatment compared to placebo [6]. In contrast to these results, other studies, such as the HYVET [7] and SPRINT [8] trials, found a clear reduction in mortality associated with BP reduction in the very elderly (older than 80 in HYVET or older than 75 in SPRINT). However, individuals included in clinical trials of antihypertensive treatment were, other than hypertension, a relatively healthy population. In contrast, we studied a cohort of patients with advanced decompensated diseases, comorbidities, and physical functional impairment. In this particular situation, the level of BP, either peripheral or aortic, was not related to 1-year mortality. Similar results were also observed by Zhang et al. [11] in a group of very elderly hospitalized patients.

We found significant increases in the SD of 24 h systolic BP in patients who died after 1 year of follow-up compared to those who remained alive. Moreover, in models adjusted for clinical confounders and the absolute value of 24 h systolic BP, a 1 SD increase in brachial systolic BP variability increased the risk of 1-year mortality by 38%. Increased BP variability in hypertension has been related to both organ damage and cardiovascular outcomes [3]. BP variability can be evaluated in the long term (between visits), mid term (day by day or week by week), and short term (repeated measurements over 24 h). In the elderly population, most studies have evaluated visit-to-visit variability (in community-living elders) [16,17] or day-by-day variability in hospitalized patients older than 75 years [15] or with acute coronary syndromes [19]. High long-term or mid-term BP variabilities have both been associated with mortality. With respect to short-term BP variability, an increased 24 h weighted SD (the mean of daytime and nighttime SD, weighted for the time duration of each period) was found to be associated with 8-year mortality in older (>65 years) participants of the Second Australian National Blood Pressure Study [18]. However, no data are available in the very elderly population regarding short-term or 24 h BP variability. Mechanisms implicated in BP variability are not completely understood, but they probably differ between long-term and short-term variabilities. Whereas the former is probably related to BP measurement conditions and techniques, as well as adherence issues, short-term BP variability is related to sympathetic control of the cardiovascular system, circadian patterns, and arterial stiffness [3]. In studies carried out in ambulatory patients, measurement of daytime, nighttime, or weighted SD is preferred over 24 h SD. However, as hospitalized patients are frequently in bed for many hours and without a clear distinction between circadian periods, we chose 24 h SD as the main measure of variability.

The relationship between increased BP variability and mortality also has therapeutic implications. Patients hospitalized with wide BP oscillations are frequently treated with short-acting antihypertensive drugs, administered depending on a punctual BP measurement. The use of such drugs might promote a more pronounced BP oscillation, thus increasing short-term BP variability. Some caution needs to be taken with the use of such treatment in very elderly hospitalized patients.

We also found an inverse correlation between heart rate variability and 1-year mortality. The SD of 24 h heart rate was significantly lower in patients who died during the 1-year follow-up. Moreover, after adjusting for clinical confounders and 24 h heart rate, 1 SD reduction in heart rate variability significantly increased the risk of mortality by 32%. Heart rate variability, measured by 24 h continuous electrocardiogram, has been inversely related with mortality and cardiovascular events in patients with cardiovascular or renal diseases [25,26,27]. A reduced heart rate variability is likely to reflect autonomic dysfunction, which is possibly related to advanced disease and loss of function. We observed that heart rate variability measured discontinuously by 24 h monitoring also relates to mortality.

The relationship between aortic stiffness and both mortality and cardiovascular outcomes has been well established in the general population, as well as in subjects at risk of cardiovascular events [4,5]. In elderly people from China and Japan, brachial ankle PWV was found to be associated with mortality [12,13], although these studies were carried out in a relatively healthy population. In patients with reduced [28] or preserved [14] congestive heart failure admitted to the hospital, increased brachial ankle PWV was also found to be associated with the development of new cardiovascular events. In contrast, Zhang et al. did not find a significant association between PWV and mortality in elderly individuals admitted to the hospital [11].

We observed that aPWV was associated with 1-year mortality in the very old, frail population with comorbidities. Compared to patients who remained alive after 1 year of follow-up, those who died presented significant increased values of aPWV. In models adjusted for clinical confounders and 24 h BP, 1 SD increase in aPWV was associated with 3.3-times more risk in terms of 1-year mortality. These results confirm the relationship between arterial stiffness and mortality in the elderly population and extend such prognostic value to the studied population. Differences between our results and those obtained by Zhang et al. [11] are possibly related to the characteristics of patients studied. In this view, we included patients who were admitted due to a decompensation of a chronic condition, with congestive heart failure as the cause of such admissions in more than 50% of patients. In addition, we obtained all the arterial parameters by computing repeated measures over 24 h (more than 70 measurements in each patient), thus increasing the robustness of each parameter.

We also observed that the BP variability ratio, another parameter probably reflecting arterial stiffness, was associated with 1-year mortality. Values were significantly higher in patients who died during follow-up, and fully adjusted models also revealed an association with mortality (31% increase in mortality for 1 SD increase in brachial BPVR). BPVR was proposed by Gavish et al. [23], as derived from the rate between systolic and diastolic 24 h SD. Although obtained from two BP variability estimates, it is considered to reflect more arterial stiffness than BPV. BPVR has been previously associated with mortality in the elderly population [29]. Our results also confirm its prognostic value extended to the very old population with decompensated chronic conditions.

The present results have obvious limitations due to the study nature. Observational studies in this population of very elderly, sick individuals pose additional difficulties in examining the prognostic value of any specific parameter added to other components of the patient’s clinical situation. Although Barthel and Charlson Indexes are well-recognized estimates of functional status and comorbidities, several other confounders not included in such scales and not evaluated in the present study may influence the outcome. Strengths of this study include a relatively homogeneous population with a narrow age window, and the many estimates obtained after 24 h monitoring, including more than 70 measurements per patient.

In conclusion, increased arterial stiffness, as measured by aPWV and BPVR, as well as short-term systolic BPV, are related to 1-year mortality in a group of very elderly patients admitted to the hospital due to a decompensation of a chronic condition. These results may indicate that such measurements (PWV and BPV) could be helpful in the prognostic evaluation of the very elderly population.

## Figures and Tables

**Table 1:** Differences in clinical parameters, BP estimates, aortic pulse wave velocity (aPWV), and blood pressure variability (BPV) between patients who died or remained alive at 1-year follow-up.

Parameter	All Patients	Alive	Dead	*p* Value
	*n* = 249	*n* = 177	*n* = 72	
Male gender	84 (33.7%)	60 (33.9%)	24 (33.3%)	0.932
Age, years	87 (3–89)	86 (82–89)	88 (85–91)	<0.001
Obesity *	69 (27.7%)	45 (25.4%)	24 (33.3%)	0.215
Hypertension	219 (88.0%)	159 (89.8%)	60 (83.3%)	0.196
Diabetes	82 (32.9%)	54 (30.5%)	28 (38.9%)	0.235
Cardiovascular disease	83 (33.3%)	58 (32.8%)	25 (34.7%)	0.769
Barthel index, cu	70 (50–95)	85 (60–100)	65 (45–70)	<0.001
Charlson index, cu	2 (2–3)	2 (1–3)	3 (2–3)	0.022
24-h brachial BP and BPV, mmHg				
24-h SBP	124.3 ± 17.1	124.4 ± 16.4	124.0 ± 18.6	0.865
24-h DBP	69.0 ± 9.6	69.1 ± 9.8	68.7 ± 9.3	0.427
SD of 24-h SBP	14.2 ± 3.7	13.5 ± 3.2	15.9 ± 4.2	<0.001
SD of 24-h DBP	9.8 ± 2.5	9.6 ± 2.3	10.0 ± 3.0	0.337
24-h aortic BP and BPV, mmHg				
24-h SBP	110.0 ± 14.5	110.2 ± 14.1	109.5 ± 15.5	0.732
24-h DBP	70.6 ± 9.8	70.7 ± 10.0	70.3 ± 9.4	0.774
SD of 24-h SBP	13.6 ± 4.1	13.1 ± 3.7	15.0 ± 4.7	0.003
SD of 24-h DBP	9.2 ± 2.2	9.1 ± 2.2	9.4 ± 2.3	0.343
BP variability ratio				
Brachial	1.51 ± 0.42	1.44 ± 0.33	1.68 ± 0.55	0.001
Aortic	1.53 ± 0.47	1.47 ± 0.39	1.66 ± 0.60	0.016
24-h HR, bpm				
24-h HR	73.1 ± 12.2	73.7 ± 11.4	71.6 ± 14.1	0.234
SD of 24-h HR	6.9 ± 3.3	7.3 ± 3.4	5.9 ± 2.8	0.003
24-h aPWV	13.3 ± 1.0	13.1 ± 1.0	13.8 ± 1.0	<0.001

Data expressed as mean ± standard deviation; median (interquartile range), or *n* (%). * Obesity defined as a body mass index ≥ 30 kg/m^2^.

**Table 2:** Hazard ratios for 1 SD change in 24 h blood pressures, 24 h heart rate, blood pressure and heart rate variabilities, aortic pulse wave velocity (aPWV), and blood pressure variability ratio (BPVR) in relation to 1-year mortality.

Parameter	Model 1	Model 2	Model 3
Blood pressure and heart rate			
24-h SBP			
Brachial	1.08 (0.86–1.36)	1.02 (0.81–1.29)	
Aortic	1.15 (0.91–1.45)	1.07 (0.86–1.35)	
24-h DBP			
Brachial	1.02 (0.81–1.28)	0.99 (0.78–1.26)	
Aortic	1.08 (0.85–1.36)	1.06 (0.86–1.35)	
24-h HR	0.92 (0.74–1.13)	0.83 (0.63–1.10)	
Blood pressure and heart rate variabilities			
SD of 24-h SBP			
Brachial	1.39 (1.12–1.74)	1.32 (1.03–1.68)	1.38 (1.06–1.80)
Aortic	1.19 (0.96–1.47)	1.14 (0.90–1.43)	1.12 (0.87–1.46)
SD of 24-h DBP			
Brachial	1.04 (0.83–1.30)	1.04 (0.79–1.36)	1.05 (0.78–1.42)
Aortic	1.07 (0.85–1.34)	1.01 (0.78–1.29)	0.99 (0.76–1.29)
SD of 24-h HR	0.70 (0.54–0.91)	0.69 (0.52–0.92)	0.68 (0.48–0.95)
Arterial stiffness			
24-h aPWV	1.63 (1.29–2.06)	1.88 (1.29–2.73)	3.30 (2.20–4.93)
BPVR			
Brachial	1.34 (1.11–1.62)	1.31 (1.06–1.62)	1.31 (1.06–1.62)
Aortic	1.17 (0.95–1.44)	1.14 (0.92–1.41)	1.13 (0.91–1.41)

Model 1 is unadjusted. Model 2 is adjusted for age, sex, obesity, diabetes, previous cardiovascular disease, Barthel Index, Charlson score, diagnosis of CHF at entry, and use of beta blockers. Model 3 is adjusted as model 2 plus the corresponding 24 h value (aPWV and BPVR were adjusted for 24 h brachial SBP).

**Table 3:** Differences in clinical parameters, BP estimates, aortic pulse wave velocity (aPWV), and blood pressure variability (BPV) in patients with or without the combined endpoint of death or readmission during the year of follow-up.

Parameter	Dead or Readmitted	Alive and Not Requiring Readmission	*p* Value
	*n* = 159	*n* = 90	
Male gender	57 (35.8%)	27 (30.0%)	0.403
Age, years	87 (84–90)	86 (83–89)	198
Obesity *	36 (22.6%)	33 (36.7%)	0.019
Hypertension	132 (83.0%)	87 (96.7%)	0.001
Diabetes	46 (28.9%)	36 (40.0%)	0.092
Cardiovascular disease	51 (32.1%)	32 (35.6%)	0.579
Barthel index, cu	70 (50–95)	70 (50–95)	0.966
Charlson score, cu	3 (2–3)	3 (1–3)	0.113
24 h brachial BP and BPV, mmHg			
24 h SBP	123.7 ± 16.9	125.3 ± 17.3	0.482
24 h DBP	68.9 ± 8.5	69.3 ± 11.4	0.773
SD of 24 h SBP	14.3 ± 3.9	13.9 ± 3.2	0.308
SD of 24 h DBP	9.6 ± 2.6	10.0 ± 2.3	0.254
24 h aortic BP and BPV, mmHg			
24 h SBP	109.2 ± 13.6	111.6 ± 15.9	0.21
24 h DBP	70.6 ± 8.7	70.7 ± 11.6	0.954
SD of 24 h SBP	13.7 ± 4.1	13.4 ± 4.1	0.547
SD of 24 h DBP	9.2 ± 2.3	9.2 ± 2.1	0.871
BP variability ratio			
Brachial	1.55 ± 0.45	1.43 ± 0.36	0.029
Aortic	1.55 ± 0.49	1.48 ± 0.43	0.285
24 h HR, bpm			
24 h HR	72.7 ± 13.1	73.8 ± 10.6	0.501
SD of 24 h HR	6.6 ± 3.2	7.3 ± 3.5	0.163
24 h aPWV	13.4 ± 1.0	13.2 ± 1.0	0.114

Data expressed as mean ± standard deviation; median (interquartile range), or *n* (%). * Obesity defined as a body mass index ≥ 30 kg/m^2^.

**Table 4:** Hazard ratios for 1 SD change of 24 h blood pressures, 24 h heart rate, blood pressure and heart rate variabilities, aortic pulse wave velocity (aPWV), and blood pressure variability ratio (BPVR) in relation to the combined endpoint of death or readmission.

Parameter	Model 1	Model 2	Model 3
Blood pressure and heart rate			
24 h SBP			
Brachial	1.06 (0.90–1.24)	0.99 (0.84–1.18)	
Aortic	1.03 (0.88–1.20)	0.98 (0.83–1.16)	
24 h DBP			
Brachial	1.01 (0.87–1.17)	0.93 (0.79–1.10)	
Aortic	1.04 (0.90–1.21)	0.98 (0.83–1.15)	
24 h HR	0.91 (0.78–1.07)	0.98 (0.82–1.18)	
Blood pressure and heart rate variabilities			
SD of 24 h SBP			
Brachial	1.01 (0.86–1.19)	1.06 (0.90–1.25)	1.07 (0.90–1.28)
Aortic	1.03 (0.89–1.20)	1.06 (0.91–1.24)	1.08 (0.91–1.28)
SD of 24-hourDBP			
Brachial	0.90 (0.77–1.06)	0.86 (0.73–1.02)	0.87 (0.72–1.04)
Aortic	0.98 (0.83–1.14)	0.99 (0.84–1.17)	0.99 (0.83–1.18)
SD of 24 h HR	0.88 (0.75–1.04)	0.88 (0.74–1.04)	0.85 (0.70–1.03)
Arterial stiffness			
24 h aPWV	1.07 (0.91–1.26)	1.39 (1.03–1.86)	2.11 (1.49–3.00)
BPVR			
Brachial	1.10 (0.96–1.27)	1.19 (1.03–1.37)	1.19 (1.03–1.38)
Aortic	1.07 (0.92–1.25)	1.12 (0.96–1.32)	1.13 (0.96–1.32)

Model 1 is unadjusted. Model 2 is adjusted for age, sex, obesity, diabetes, previous cardiovascular disease, Barthel Index, Charlson score, diagnosis of CHF at entry, and use of beta blockers. Model 3 is adjusted as model 2 plus the corresponding 24 h value (aPWV and BPVR were adjusted for 24 h brachial SBP).

## Data Availability

The data presented in this study are available on request from the corresponding author. The data are not publicly available due to patient privacy.

## Supplementary Materials

The following supporting information can be downloaded at: https://www.mdpi.com/article/10.3390/jcm12041510/s1. Table S1: Hazard ratios for 1 SD change of 24-h blood pressures, 24-h heart rate, blood pressure and heart rate variabilities, aortic pulse wave velocity and blood pressure variability ratio in relation to death in subjects younger or older than 85 years. Table S2: Hazard ratios for 1 SD change of 24-h blood pressures, 24-h heart rate, blood pressure and heart rate variabilities, aortic pulse wave velocity and blood pressure variability ratio in relation to death in men and women. Table S3: Hazard ratios for 1 SD change of 24-h blood pressures, 24-h heart rate, blood pressure and heart rate variabilities, aortic pulse wave velocity (aPWV) and blood pressure variability ratio in relation to death in diabetic and non diabetic patients. Table S4: Hazard ratios for 1 SD change of 24-h blood pressures, 24-h heart rate, blood pressure and heart rate variabilities, aortic pulse wave velocity (aPWV) and blood pressure variability ratio in relation to death in patients with a Barthel index lower or greater than 70. Table S5: Hazard ratios for 1 SD change of 24-h blood pressures, 24-h heart rate, blood pressure and heart rate variabilities, aortic pulse wave velocity (aPWV) and blood pressure variability ratio in relation to death in patients with congestive heart failure (CHF) or other types of chronic disease decompensation.
