# Supplementary material for: Pulse Wave Velocity and Blood Pressure Variability as Prognostic Indicators in Very Elderly Patients
Source: J Clin Med. 2023 Feb 14;12(4):1510. doi: 10.3390/jcm12041510 (PMC9963376; doi:10.3390/jcm12041510)
Supplement: Supplementary file 1 [file jcm-12-01510-s001.zip › jcm-2183593-supplementary.pdf]

Pulse Wave Velocity and Blood Pressure Variability as Prognostic Indicators in Very Elderly Patients

Alejandro de la Sierra, Cristina Sierra, Marcos Murillo, Tomasso F. Aiello, Aina Mateu and Pedro Almagro [1]

Supplementary Tables

Table S1. Hazard ratios for 1 SD change of 24-hour blood pressures, 24-hour heart rate, blood pressure and heart rate variabilities, aortic pulse wave velocity and blood pressure variability ratio in relation to death in subjects younger or older than 85 years

Parameter	≤85 years<br>N=101; 20 deaths	> 85 years<br>N=148; 52 deaths
**Blood pressure and heart rate**		
24-hour SBP		
Brachial	1.02 (0.63-1.65)	1.38 (0.98-1.95)
Aortic	1.17 (0.74-1.83)	1.47 (1.02-2.12)
24-hour DBP		
Brachial	1.11 (0.72-1.72)	1.38 (0.95-2.02)
Aortic	1.14 (0.73-1.78)	1.49 (1.03-2.17)
24-hour HR	1.04 (0.69-1.94)	0.43 (0.28-0.68)
**Blood pressure and heart rate variability**		
SD of 24-hour SBP		
Brachial	1.41 (0.80-2.47)	1.34 (0.94-1.91)
Aortic	1.02 (0.57-1.80)	1.11 (0.79-1.57)
SD of 24-hourDBP		
Brachial	1.26 (0.84-1.90)	0.86 (0.54-1.37)
Aortic	0.95 (0.59-1.51)	0.99 (0.70-1.41)
SD of 24-hour HR	0.36 (0.18-0.74)	0.94 (0.55-1.59)
**Arterial stiffness**		
24-hour aPWV	3.85 (1.88-7.87)	2.61 (1.36-5.01)
BPVR		
Brachial	1.20 (0.76-1.90)	1.26 (0.97-1.63)
Aortic	1.11 (0.66-1.87)	1.08 (0.84-1.40)

Model adjusted for sex, obesity, diabetes, previous cardiovascular disease, Barthel index, Charlson score, diagnosis of CHF at entry and use of beta-blockers. Additional adjustments for brachial or central 24-h systolic BP (SD of 24-h SBP, 24-h aPWV and BPVR), for brachial or central 24-h diastolic BP (SD of 24-h DBP) or 24-h HR (SD of 24-h HR)

Table S2. Hazard ratios for 1 SD change of 24-hour blood pressures, 24-hour heart rate, blood pressure and heart rate variabilities, aortic pulse wave velocity and blood pressure variability ratio in relation to death in men and women

**Parameter**	**Men**<br>**N=84; 24 deaths**	**Women**<br>**N=165; 48 deaths**
	**Blood pressure and heart rate**	
24-hour SBP		
Brachial	0.91 (0.59-1.38)	1.14 (0.85-1.53)
Aortic	1.11 (0.70-1.75)	1.12 (0.85-1.47)
24-hour DBP		
Brachial	1.41 (0.87-2.27)	0.98 (0.73-1.32)
Aortic	1.78 (1.04-3.04)	0.99 (0.74-1.34)
24-hour HR	0.98 (0.60-1.61)	0.64 (0.40-1.05)
	**Blood pressure and heart rate variability**	
SD of 24-hour SBP		
Brachial	1.08 (0.68-1.72)	1.87 (1.34-2.61)
Aortic	0.77 (0.47-1.25)	1.50 (1.05-2.14)
SD of 24-hourDBP		
Brachial	0.58 (0.34-0.97)	1.94 (1.31-2.86)
Aortic	0.94 (0.59-1.49)	1.12 (0.80-1.56)
SD of 24-hour HR	0.34 (0.17-0.67)	0.80 (0.52-1.22)
	**Arterial stiffness**	
24-hour aPWV	1.84 (0.77-4.44)	3.38 (1.85-6.18)
BPVR		
Brachial	1.54 (1.09-2.18)	1.23 (0.90-1.69)
Aortic	0.91 (0.56-1.49)	1.22 (0.93-1.60)

Model adjusted for age, obesity, diabetes, previous cardiovascular disease, Barthel index, Charlson score, diagnosis of CHF at entry and use of beta-blockers. Additional adjustments for brachial or central 24-h systolic BP (SD of 24-h SBP, 24-h aPWV and BPVR), for brachial or central 24-h diastolic BP (SD of 24-h DBP) or 24-h HR (SD of 24-h HR)

Table S3. Hazard ratios for 1 SD change of 24-hour blood pressures, 24-hour heart rate, blood pressure and heart rate variabilities, aortic pulse wave velocity (aPWV) and blood pressure variability ratio in relation to death in diabetic and non diabetic patients

**Parameter**	**With diabetis N=82; 28 deaths**	**Without diabetis N=167; 44 deaths**
**Blood pressure and heart rate**		
24-hour SBP		
Brachial	1.07 (0.67-1.73)	1.26 (0.89-1.79)
Aortic	1.04 (0.68-1.59)	1.41 (0.98-2.03)
24-hour DBP		
Brachial	0.81 (0.50-1.31)	1.39 (0.92-2.12)
Aortic	0.88 (0.55-1.41)	1.48 (0.97-2.25)
24-hour HR	0.17 (0.07-0.40)	0.89 (0.62-1.28)
**Blood pressure and heart rate variability**		
SD of 24-hour SBP		
Brachial	1.48 (0.74-2.99)	1.16 (0.80-1.67)
Aortic	1.27 (0.72-2.23)	1.01 (0.70-1.44)
SD of 24-hourDBP		
Brachial	1.13 (0.56-2.27)	1.04 (0.72-1.52)
Aortic	0.96 (0.63-1.48)	0.95 (0.66-1.38)
SD of 24-hour HR	0.36 (0.15-0.90)	0.75 (0.52-1.10)
**Arterial stiffness**		
24-hour aPWV	2.74 (1.17-6.42)	3.46 (1.78-6.73)
BPVR		
Brachial	1.26 (0.78-2.02)	1.21 (0.89-1.65)
Aortic	1.15 (0.81-1.64)	1.04 (0.72-1.49)

Model adjusted for age, sex, obesity, previous cardiovascular disease, Barthel index, Charlson score, diagnosis of CHF at entry and use of beta-blockers. Additional adjustments for brachial or central 24-h systolic BP (SD of 24-h SBP, 24-h aPWV and BPVR), for brachial or central 24-h diastolic BP (SD of 24-h DBP) or 24-h HR (SD of 24-h HR)

Table S4. Hazard ratios for 1 SD change of 24-hour blood pressures, 24-hour heart rate, blood pressure and heart rate variabilities, aortic pulse wave velocity (aPWV) and blood pressure variability ratio in relation to death in patients with a Barthel index lower or greater than 70

Parameter	Barthel < 70<br>N=110; 41 deaths	Barthel ≥ 70<br>N=139; 31 deaths
**Blood pressure and heart rate**		
24-hour SBP		
Brachial	1.28 (0.89-1.85)	1.12 (0.74-1.68)
Aortic	1.17 (0.83-1.65)	1.32 (0.88-1.97)
24-hour DBP		
Brachial	0.91 (0.61-1.34)	1.44 (0.92-2.25)
Aortic	1.03 (0.75-1.42)	1.65 (1.04-2.63)
24-hour HR	0.30 (0.14-0.64)	0.97 (0.66-1.42)
**Blood pressure and heart rate variability**		
SD of 24-hour SBP		
Brachial	1.82 (1.27-2.59)	1.21 (0.79-1.87)
Aortic	1.64 (1.13-2.38)	0.88 (0.55-1.39)
SD of 24-hourDBP		
Brachial	1.67 (1.05-2.64)	1.05 (0.73-1.50)
Aortic	1.15 (0.81-1.64)	1.10 (0.72-1.68)
SD of 24-hour HR	0.57 (0.25-1.32)	0.80 (0.50-1.26)
**Arterial stiffness**		
24-hour aPWV	3.52 (1.77-7.02)	2.73 (1.32-5.66)
BPVR		
Brachial	1.35 (1.01-1.80)	1.21 (0.85-1.71)
Aortic	1.19 (0.91-1.55)	0.89 (0.54-1.47)

Model adjusted for age, sex, obesity, diabetes, previous cardiovascular disease, Charlson score, diagnosis of CHF at entry and use of beta-blockers. Additional adjustments for brachial or central 24-h systolic BP (SD of 24-h SBP, 24-h aPWV and BPVR), for brachial or central 24-h diastolic BP (SD of 24-h DBP) or 24-h HR (SD of 24-h HR)

Table S5. Hazard ratios for 1 SD change of 24-hour blood pressures, 24-hour heart rate, blood pressure and heart rate variabilities, aortic pulse wave velocity (aPWV) and blood pressure variability ratio in relation to death in patients with congestive heart failure (CHF) or other types of chronic disease decompensation

**Parameter**	**With CHF**<br>**N=149; 42 deaths**	**Without CHF**<br>**N=100; 30 deaths**
	**Blood pressure and heart rate**	
24-hour SBP		
Brachial	0.86 (0.62-1.21)	1.32 (0.86-2.03)
Aortic	0.96 (0.69-1.34)	1.48 (0.92-2.39)
24-hour DBP		
Brachial	0.95 (0.68-1.33)	1.53 (0.92-2.54)
Aortic	0.95 (0.67-1.33)	1.81 (1.08-3.04)
24-hour HR	0.90 (0.59-1.37)	0.82 (0.50-1.36)
	**Blood pressure and heart rate variability**	
SD of 24-hour SBP		
Brachial	1.68 (1.20-2.34)	0.91 (0.57-1.47)
Aortic	1.33 (0.93-1.90)	0.90 (0.59-1.35)
SD of 24-hourDBP		
Brachial	1.31 (0.91-1.89)	0.64 (0.37-1.09)
Aortic	1.42 (0.98-2.04)	0.65 (0.39-1.08)
SD of 24-hour HR	0.68 (0.44-1.06)	0.44 (0.24-0.80)
	**Arterial stiffness**	
24-hour aPWV	2.59 (1.61-4.18)	8.95 (2.63-30.4)
BPVR		
Brachial	1.25 (0.94-1.67)	1.47 (0.91-2.37)
Aortic	1.05 (0.70-1.57)	1.18 (0.84-1.67)

Model adjusted for age, sex, obesity, diabetes, previous cardiovascular disease, Barthel index, Charlson score, and use of beta blockers. Additional adjustments for brachial or central 24-h systolic BP (SD of 24-h SBP, 24-h aPWV and BPVR), for brachial or central 24-h diastolic BP (SD of 24-h DBP) or 24-h HR (SD of 24-h HR)
